# Withdrawal of Clinically Assisted Nutrition and Hydration for a Patient in a Persistent Vegetative State: A Report of a Case in the UK With Cross-Jurisdictional and Islamic Law Perspectives

**DOI:** 10.7759/cureus.110880

**Published:** 2026-06-15

**Authors:** Sugna Divakar Prabhu, Hussain Mahmood, Sarah Menzies

**Affiliations:** 1 Geriatrics, Wexham Park Hospital, Slough, GBR; 2 Palliative care, Wexham Park Hospital, Slough, GBR; 3 Respiratory Medicine, Wexham Park Hospital, Slough, GBR

**Keywords:** advance directive, bma/rcp guideline on canh, clinically assisted nutrition and hydration in vegetative state, islamic bioethics, uk law

## Abstract

The withdrawal of clinically assisted nutrition and hydration (CANH) is a legally and ethically complex intervention governed by distinct frameworks across the world. In the UK, the British Medical Association (BMA) and Royal College of Physicians (RCP) guideline on CANH, formulated in 2018 after a Supreme Court ruling, provides a framework for decisions. In contrast, Islamic law treats nutritional support as basic care, and its withdrawal is prohibited unless it causes direct harm or there is the presence of an advance directive. Cross-jurisdictional cases where a patient moves between these legal frameworks are rarely described in published literature.

We report the case of a 61-year-old woman with no co-morbidities who was in Qatar in November 2024 when she had an out-of-hospital cardiac arrest, resulting in a hypoxic brain injury and later a persistent vegetative state. Her family requested withdrawal of care, citing her wishes to avoid living with a disability. The hospital consulted its ethical and legal departments and concluded that they could not withdraw nutrition as there was no prior explicit directive from the patient. She was repatriated to her local hospital in the UK after eight weeks. She remained in a vegetative state, and her brain imaging was unchanged. Her care team followed the BMA/RCP CANH 2024 guidelines to assist with the process of withdrawing nutrition and hydration and obtained further specialist and legal team opinions. Letters of support from family and friends were also collected. Supportive treatment was withdrawn nine days after UK repatriation, and she died three weeks later with palliative team input.

This case illustrates the practical challenges of applying CANH withdrawal guidelines across jurisdictions with different legal and ethical frameworks. It demonstrates the importance of a robust, workable framework like the BMA/RCP guideline on withdrawal of CANH. It highlights the value of advance directives, close family involvement, and legal framework in clinical decisions. Clinicians caring for Muslim patients should be aware of the Islamic bioethical perspective on CANH, which may shape family expectations and discussions in similar situations.

## Introduction

Clinically assisted nutrition and hydration (CANH) is a form of medical treatment where artificial feed is given to the patient in the form of tube-feeding (e.g., via nasogastric tube, percutaneous endoscopic gastrostomy (PEG), or parenteral nutrition) [1]. The withdrawal of CANH has been a subject of sustained legal and ethical scrutiny in the United Kingdom. Starting in 1993, it was established that CANH is a treatment and not routine care [2]. Subsequent cases progressively refined the criteria and processes applicable to such decisions [3-5]. In 2018, the Supreme Court concluded that there was no legal requirement for cases to be brought to the court [6], provided there was agreement upon what was in the patient's best interests. The British Medical Association (BMA)/Royal College of Physicians (RCP) CANH guidelines were initially written in 2018, with a recent update in 2024 [1].

Cases in which patients are repatriated to the UK specifically to access the legal framework for CANH withdrawal are rare. In Islamic law, CANH is considered basic care rather than treatment [7]. As the patient was in two different countries where CANH is governed by different laws, her case provides insight into this. We describe how the BMA/RCP 2024 guidelines were applied following repatriation and discuss the differences in the legal frameworks across the two countries. We also outline the Islamic bioethical perspective of CANH [8,9], which is of relevance to clinicians caring for Muslim patients in the UK.

## Case presentation

A 61-year-old woman with no known co-morbidities visited her husband in Qatar in early November 2024, where she had an out-of-hospital cardiac arrest with immediate resuscitation by the emergency services. The initial ECG showed ST elevation in the anterior leads, and percutaneous coronary intervention (PCI) to the left anterior descending artery (LAD) was performed. Due to a low Glasgow Coma Scale [10], she remained intubated, and a tracheostomy was performed. Over the eight weeks following admission, there was no significant improvement in her neurological response, and brain CT and MRI scans demonstrated hypoxic-ischemic encephalopathy.

Her family was aware of the BMA/RCP guidelines and requested withdrawal of CANH [1], citing her wishes to avoid living in a vegetative state. Her case was reviewed by the hospital’s legal and ethical committees, who concluded that they could not withdraw this life-sustaining treatment as she had not previously completed an advanced directive. They did establish her Do Not Attempt Resuscitation (DNAR) status; however, she continued to receive supportive care, including fluids and nasogastric feeding.

Her family was keen for her to be repatriated to the UK for withdrawal of CANH, and she was transferred to her local hospital eight weeks after the cardiac event. Her Glasgow Coma Scale [10] was 8 on admission (eyes 4, verbal 1, motor 3) and did not improve. From a cardiac standpoint, her initial echo from Qatar showed a left ventricular (LV) ejection fraction of 38%, which subsequently improved to 47%. She was commenced on dual antiplatelets and a beta blocker. Routine blood tests were unremarkable apart from a low potassium initially (Table 1), and her chest X-ray was normal (Figure 1). Her electroencephalogram (EEG) reported no electro-cortical activity, and her repeat MRI brain confirmed findings of extensive diffuse hypoxic ischemic encephalopathy (Figure 2). Formal assessment using the Coma Recovery Scale-Revised (CRS-R) [11] was performed by the neurophysiology team, wherein she scored below 8, indicating vegetative state. Taken together, the absence of electro-cortical activity, MRI findings, CRS-R score, and review from the Neurology team supported a diagnosis of persistent vegetative state with a poor prognosis for a meaningful recovery.

**Table 1:** Initial blood tests in the UK ↑ indicates an increase in value, ↓indicates a decrease in value. eGFR, estimated glomerular filtration rate; CKD-EPI, Chronic Kidney Disease Epidemiology Collaboration; CK, creatine kinase; CRP, C-reactive protein; WBC, white blood cells; RBC, red blood cells; MCV, mean corpuscular volume; MCH, mean corpuscular hemoglobin; MCHC, mean corpuscular hemoglobin concentration; RDW, red cell distribution width; MPV, mean platelet volume.

Test	26/12/24 04:30	27/12/24 09:29	Reference range (units)
Sodium	149 ↑	143	133-146 mmol/L
Potassium	2.6 ↓	2.5 ↓	3.5-5.3 mmol/L
Urea	10.5 ↑	6.0	2.5-7.8 mmol/L
Creatinine	40 ↓	24 ↓	45-90 µmol/L (F)/60-110 (M)
eGFR (CKD-EPI)	>90	>90	>90 mL/min/1.73 m²
Total bilirubin	-	7	<21 µmol/L
Alkaline phosphatase	-	113	30-130 U/L
Alanine transaminase	-	72 ↑	<40 U/L
CK	64	-	25-200 U/L (varies by sex)
Calcium	-	2.26	2.20-2.60 mmol/L
Adjusted calcium	-	2.45	2.20-2.60 mmol/L
Phosphate	-	1.15	0.80-1.50 mmol/L
Magnesium	1.17 ↑	1.03 ↑	0.70-1.00 mmol/L
CRP	8.7 ↑	17 ↑	<5 mg/L
Albumin	-	28 ↓	35-50 g/L
Hemoglobin	114 ↓	113 ↓	115-165 g/L (F)/130-180 (M)
WBC	8.4	8.2	4.0-11.0 ×10⁹/L
Platelets	383	313	150-450 ×10⁹/L
RBC	3.79	3.83	3.8-5.8 ×10¹²/L
Hematocrit	0.368 ↓	0.358 ↓	0.37-0.47 (F)/0.40-0.54 (M)
MCV	97.1	93.6	80-100 fL
MCH	30.1	29.6	27-32 pg
MCHC	310 ↓	316 ↓	320-360 g/L
RDW	14.5	14.0	11.5-14.5%
MPV	11.1	11.2	7.5-11.5 fL
Neutrophils (abs)	6.1	5.7	2.0-7.5 ×10⁹/L
Lymphocytes (abs)	1.6	1.8	1.0-4.0 ×10⁹/L
Eosinophils (abs)	0.0	0.1	0.0-0.4 ×10⁹/L

**Figure 1:**
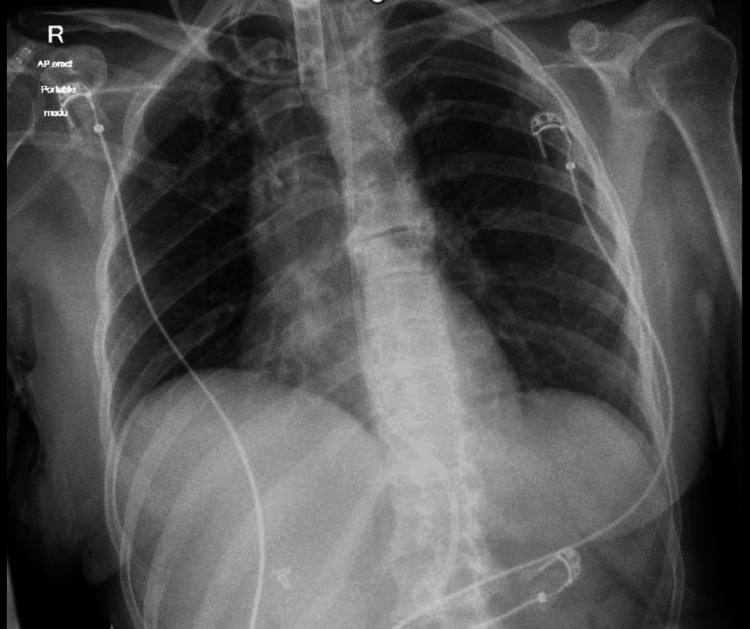
Chest X-ray showing no abnormalities

**Figure 2:**
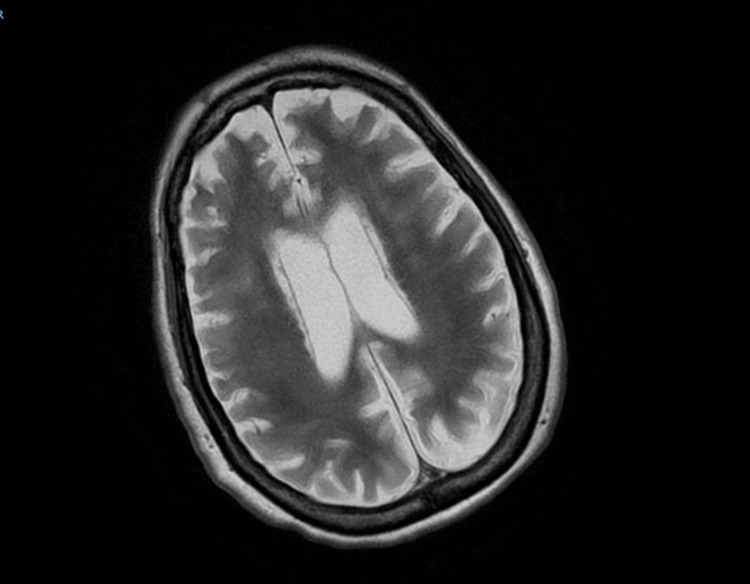
MRI brain FLAIR sequence showing diffuse hyperintense signal (bright area) consistent with hypoxic brain injury FLAIR, fluid-attenuated inversion recovery.

Her family requested withdrawal of CANH, expressing that she would not have wanted to live in her current state. Her medical team followed the BMA/RCP 2024 guidelines on withdrawal of CANH, and independent opinions were sought from two consultants and consultants in palliative care and neurology. The Trust’s legal team was also consulted. Best-interest meetings were arranged between the patient’s family and the medical team, and letters of support were collected from other close family and friends as evidence of her likely wishes. Following these investigations and consultations, it was concluded that she was in a persistent vegetative state with poor prognosis, and that it was in her best interest for CANH to be withdrawn. This decision was made nine days after her UK repatriation. She received ongoing palliative care team input and died peacefully three weeks later.

## Discussion

Cross-jurisdiction comparison: Qatar and the United Kingdom

In Qatar, withdrawal of CANH could not be authorized as our patient had not previously documented her wish to be allowed to die in the event of her suffering an event leading to significant disability. This reflects the influence of Islamic law on healthcare ethics in Qatar, where nutritional support is generally regarded as basic care rather than medical treatment.

The law is centered around preventing harm and avoiding injury, and withdrawal of CANH is permissible only if it is causing demonstrable harm or if the patient has previously expressed such a wish by way of an advance directive [7].

In the UK, the first legal foundation for CANH withdrawal was established in 1993 in the case of Anthony Bland, who had sustained crush injuries in 1989 at Hillsborough Stadium during a Liverpool Football Club match. He had brain anoxia with progression to a persistent vegetative state. His trial concluded that his CANH could be withdrawn without doctors being accused of causing harm, as meaningful neurological recovery was impossible. This outcome was upheld on appeal [5]. At that time, court authorization was deemed necessary for all such decisions.

In 2011, withdrawal of CANH was not granted for Mrs M in the court as she was still experiencing pain and having positive experiences like hearing music, and was stated to be in a minimally conscious state [2]. In 2018, Mr Y, an active man in his fifties, had a cardiac arrest leading to a hypoxic brain injury, and both his clinical team and family agreed that it would be in his best interest for CANH to be withdrawn. Following a court hearing, the Supreme Court ruled that there was no legal requirement for cases like this to be brought to the court in the future, provided there was agreement between the clinicians and the patient’s next of kin and that the Mental Capacity Act 2005 was followed and the relevant guidance observed [4].

The BMA and RCP released a guide on withdrawal of CANH in 2018, covering adults who lack the capacity to consent across a range of clinical contexts, including neurodegenerative conditions, brain injury, multiple comorbidities, or frailty, which is likely to shorten life expectancy [1]. It does not cover decisions in patients where CANH was not clinically indicated, those that were expected to die within hours or days, and cases where a decision not to provide or continue CANH was incidental to the decision to stop other life-sustaining treatments. This guidance was updated in 2024.

Islamic bioethical perspectives and relevance to UK clinical practice

Although our patient was not Muslim, and the Islamic legal framework was not directly applicable to her care in the UK, an understanding of this perspective is of practical value for clinicians in the UK who may encounter Muslim patients or families in similar situations. 

Mohiuddin et al. reviewed the previous Islamic rulings to attempt to define when withdrawal of life support could be permitted and found that the definition of futility of treatment had been inconsistent, preventing the development of a unified policy [8]. An Islamic bioethical framework for withholding and withdrawing life-sustaining treatment has been theorized using Islamic law as guiding principles, taking both length and quality of life into account (Figure 3) [9]. Such a framework may assist clinicians in facilitating discussions with Muslim patients and families facing these decisions in the UK context. 

**Figure 3:**
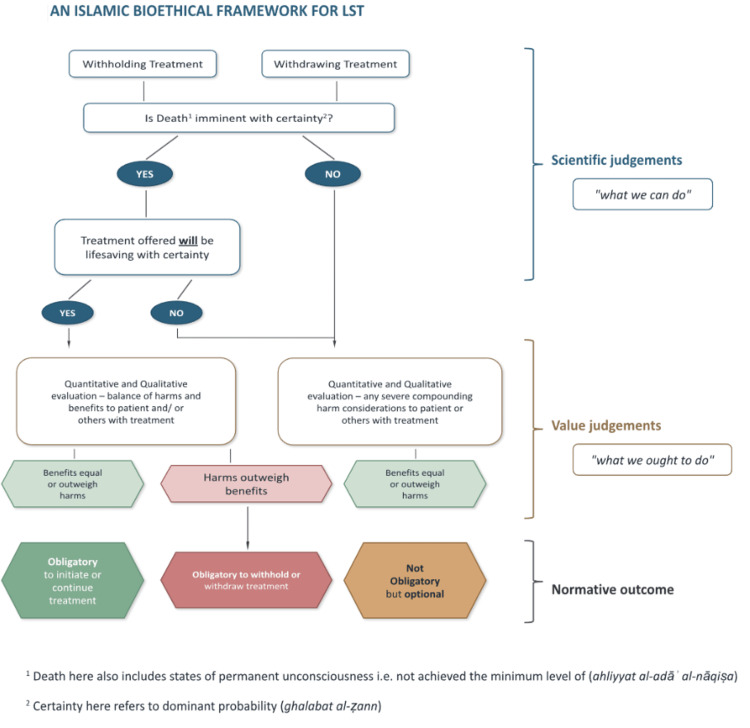
Pathway from an Islamic bioethical framework for withholding and withdrawing life-sustaining treatment Source: [9], used under the CC BY-NC 4.0 deed.

Limitations

This is a single-case report, and the findings cannot be generalized. The determination of our patient’s likely wish was based entirely on family and friends' testimony, as there was no advance directive. We acknowledge that our account of the Qatari legal process is based on information provided by the treating team and the family, and may not fully capture the nuances of Qatar’s ethical and legal framework. 

## Conclusions

This case emphasizes the value of clear, actionable guidelines in facilitating the complex process of withdrawing CANH, particularly in patients who have not documented an advanced directive. The UK guidelines provided a workable framework even in a complex cross-jurisdictional situation, enabling a timely, dignified, and legally sound outcome.

Practical lessons from the case arise. First, clinicians and the public should be encouraged to complete advance directives specifying their wishes regarding life-sustaining treatments, as this is the most effective protection of patient autonomy across different legal contexts. Second, clinicians across the world should be aware of the Islamic bioethical framework on CANH, which may shape family expectations and discussions when caring for Muslim patients.
